# Classifying motion states from neural activity of non-human primates for brain-computer interfaces

**DOI:** 10.3389/fnins.2026.1714738

**Published:** 2026-02-20

**Authors:** Yicong Xiao, Spencer Kellis, Christopher F. Reiche, Florian Solzbacher

**Affiliations:** 1 Department of Electrical and Computer Engineering, University of Utah, Salt Lake City, UT, United States; 2 Blackrock Neurotech, Salt Lake City, UT, United States; 3 Neurorestoration Center, Keck School of Medicine of USC, Los Angeles, CA, United States; 4 Department of Engineering Sciences, Jade Hochschule - University of Applied Sciences, Wilhelmshaven, Germany; 5 Department of Materials Science & Engineering, University of Utah, Salt Lake City, UT, United States; 6 Department of Biomedical Engineering, University of Utah, Salt Lake City, UT, United States

**Keywords:** brain-computer interface, correlation analysis, motion states, neural activity, offline analysis, principal component analysis, support vector machine

## Abstract

**Introduction:**

Brain-computer interface (BCI) systems commonly decode neural activity from sensorimotor areas to generate continuous control signals for cursors, robotic limbs, or other effectors. Although these decoders perform well during intended movement, neural activity persists during periods of intended non-movement, which can lead to unintended effector activation and reduced control stability. Accurately identifying intended stationary states therefore represents a key component for achieving stable and reliable BCI control.

**Methods:**

We propose a neural-state classification framework (cpSVM) that distinguishes stationary and movement states directly from intracortical neural activity. This model combines principal component analysis, correlation-based feature selection, and a linear support vector machine classifier. Offline evaluations were performed using multi-unit recordings from the premotor and primary motor cortices of two non-human primates during a center-out cursor task. Performance was compared against a conventional kinematics-based threshold-crossing method.

**Results:**

Correlation-informed dimensionality reduction revealed a clear low-dimensional separation between stationary and movement states, supporting the selection of task-relevant neural features. The cpSVM achieved high classification performance, with mean accuracies of 0.936 and 0.930 across the two subjects. Compared with the threshold-crossing method, the cpSVM consistently improved accuracy, sensitivity, specificity, and *F*-score, while substantially reducing spurious state transitions and improving output continuity.

**Discussion:**

These findings demonstrate that stationary and movement states can be reliably distinguished from intracortical neural signals using a low-dimensional, correlation-informed classification approach. The proposed framework provides a promising strategy to suppress unintended effector activation and improve continuity and stability in BCI control systems.

## Graphical Abstract

**Figure:**
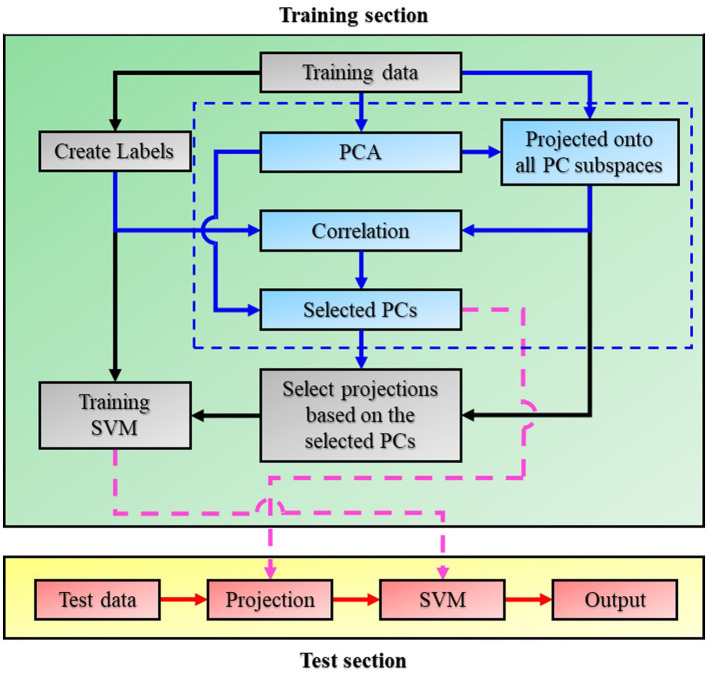
The flow chart of the new model (cpSVM). Dash frame is the flow chart of the cPCA. The figure is divided into a training section (top green box) and a test section (bottom yellow box). In the training section, the blue dashed frame highlights the cPCA procedure used to select task-relevant principal components (PCs). The blue boxes and blue arrows represent the PCA and correlation steps that identify these PCs. Black arrows indicate the process in which the selected PC projections and labels are used to train the SVM classifier. The purple dashed arrows illustrate the dependencies between the training and test sections: (1) test data are projected onto the PC subspace selected during training, and (2) the trained SVM model is applied to these projections to generate predictions. The red arrows depict the complete test pipeline.

## 1 Introduction

Brain-computer interfaces (BCI) create pathways from the brain for control of, and in some cases feedback from, external effectors (Tam et al., 2019). Established BCI methods have successfully demonstrated applications that can help paralyzed individuals gain independence, such as controlling a computer cursor (Dethier et al., 2013; Gilja et al., 2015, 2012; Sussillo et al., 2012) or a prosthetic limb (Cajigas et al., 2021; Chestek et al., 2013; Collinger et al., 2013; Schulman, 2009; Zhou et al., 2007), or even maneuvering a motorized wheelchair (Espiritu et al., 2019; Lahane et al., 2018; Tang et al., 2018). Some BCI systems decode motor activity from the brain and then recode these motor commands as control signals for the user's own limbs (Milosevic et al., 2020a,b; Osuagwu et al., 2016) or to manipulate rehabilitation devices (Bhagat et al., 2020; Cheng et al., 2020; Robinson et al., 2021; Wu et al., 2020) for recovery.

The increasing performance of BCIs clearly demonstrates the ability to decode intended movement from neural activity. However, a known drawback arises in the periods without intended movement: since brain activity never fully ceases, implanted electrodes still record spiking activity, leading the decoder to output unintended motions (e.g., erratic cursor movements) that degrade user experience. This phenomenon has been reported in prior BCI studies, where random neural fluctuations during idle periods can lead to false-positive activations (Fazli et al., 2009; Williams et al., 2013).

To address the false activation problem, previous studies explored adding a dedicated classification stage before the main movement decoder. Fazli et al. (2009) used EEG features with a probabilistic model to jointly classify multiple movement classes and a rest class, integrating the result to suppress unintended cursor motion; Williams et al. (2013) applied an L1-regularized model to ECoG features for binary task/rest detection, using the output as a control switch to enable or disable the movement decoder; Velliste et al. (2014) systematically examined the firing properties of M1 neurons during arm-rest periods in a reaching task and trained a Linear Discriminant Analysis classifier to predict arm rest states, showing that structured and discriminable neural patterns persist even in the absence of arm movement; Williams et al. (2016) used intracortical single-unit activity and local field potentials to build a population-level classifier, enabling reliable distinction between idle states and task-activated neural states. These approaches illustrate that pre-decoder classification can both prevent unintended outputs and prepare cleaner, more informative neural features for subsequent decoding.

However, most aforementioned studies were conducted under relatively idealized idle conditions, in which both behavior and neural activity corresponded to a clear resting state. In practical BCI scenarios, differences in behavior and neural activity may not be as clear. For example, preparatory neural activity may emerge in some motor-related brain regions before the onset of movement. This preparatory activity might contain information about upcoming movement but should not produce effector movement itself. However, the numerical dependence of decoder outputs on decoder inputs means that that these modulating decoder inputs will propagate changes into the outputs, potentially causing erroneous effector activation, unintended displacement, or control instability. Therefore, distinguishing intended effector stationary state, which may include not only idle periods but also preparatory neural activity, from intended effector movement state is critical for reliable BCI control.

This work, using intracortical multi-unit activity from two non-human primates (NHPs), develops a pre-decoder classification framework to distinguish between stationary and movement states during cursor control tasks. Guided by analysis of the neural data that probes how multi-units behave across stationary and movement, we incorporate dimensionality reduction and correlation analysis to inform feature selection and reduce model complexity, methods shown to improve decoding accuracy and computational efficiency (Blankertz et al., 2006; Chao et al., 2010; Schalk et al., 2004). This framework is intended to enable the decoder to be selectively activated only during intentional control, thereby reducing false positives in stationary states.

## 2 Methods

### 2.1 Data and analysis tool

Multi-unit data from NHP premotor and motor cortex (rhesus monkeys J and N), and cursor displacement information, were shared by Churchland et al. (2012) and Kaufman et al. (2015). Each NHP was implanted with one 96-electrode array in the dorsal premotor cortex (PMd, array A) and one in the primary motor cortex (M1, array B), for a total of 192 electrodes (channels).

NHPs J and N performed a direct delay center-out reaching task, with a total of 1,533 trials (J) and 1,530 trials (N). In each trial, the NHP first held the cursor at a central fixation point for at least 700 ms, triggering the appearance of a target in one of nine random directions. A variable preparatory delay period (0–1 s), during which the NHP still had to maintain the cursor at the central fixation point. Subsequently, the go cue was presented, prompting the NHP to initiate cursor movement toward the target. Successful and timely acquisition of the target, maintained at least 450 ms (J) or 700 ms (N), resulted in a juice reward. Neural signals and cursor kinematics were sampled at 1 kHz, and all analyses were performed at this temporal resolution unless otherwise specified.

For each NHP, the data from PMd and M1 were aggregated (192 channels), and the firing rate for each channel was calculated by Gaussian convolution (mean = 0 ms, SD = 3 ms) with spikes. All decoding, encoding, and analysis were performed using MATLAB (version R2022a, The MathWorks, Inc., Natick, MA) on a computer equipped with an Intel^®^ Core™ i9-10900K CPU (3.70 GHz) and an NVIDIA GeForce RTX 2080 SUPER GPU.

### 2.2 Reference labels for each trial

In this study, ground truth stationary and movement states were defined strictly at the behavioral level. The period from the initiation of cursor motion to target acquisition was defined as the behavioral movement state, whereas all other periods were treated as the behavioral stationary state. Reflecting the absence of movement at the behavioral level, the stationary state included the preparatory period where the cursor remained at the central fixation point.

**Figure 1:**
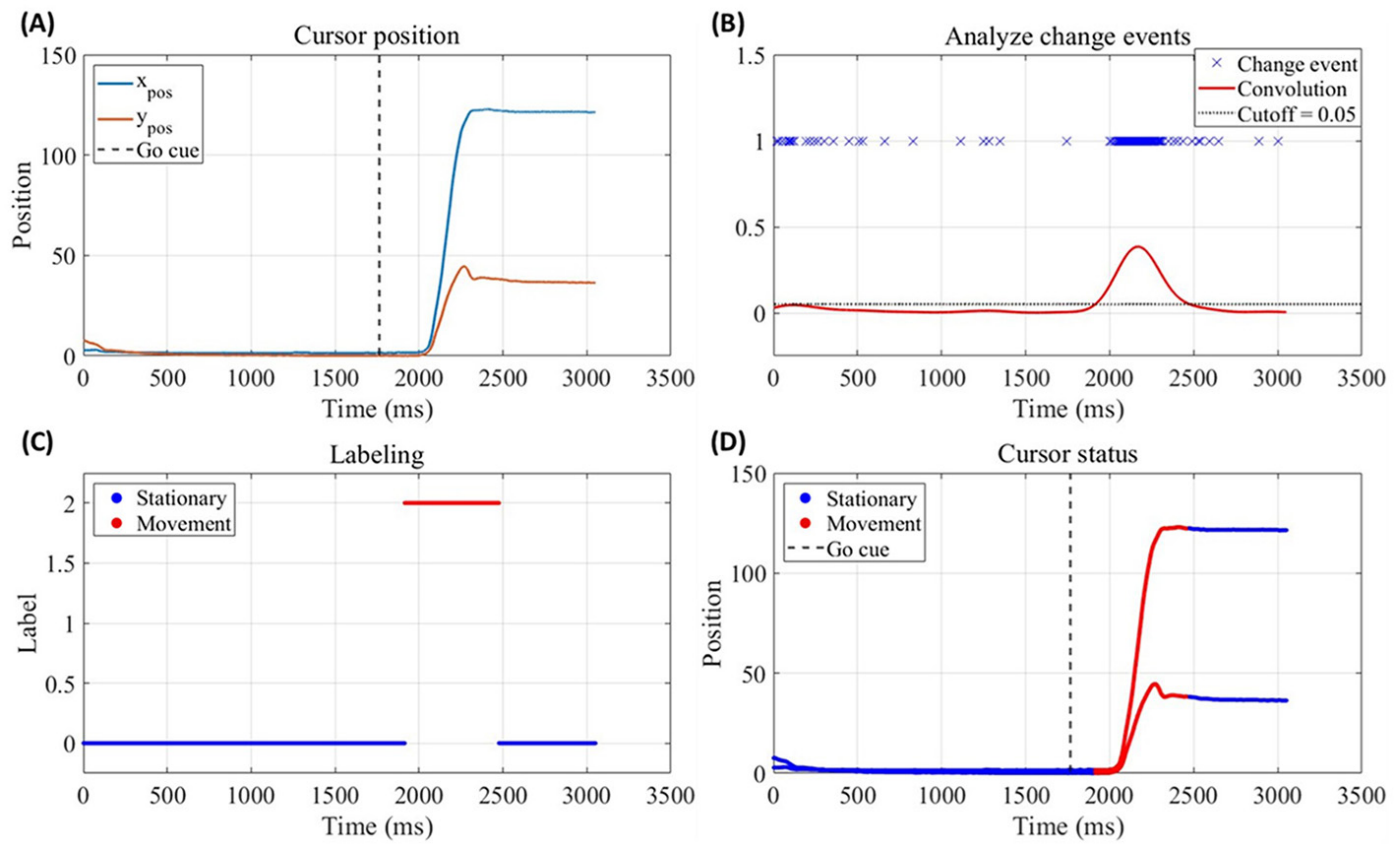
Example of reference label generation process, shown for Trial 1 of NHP J. **(A)** Cursor position data (x- and y-components over time), with vertical dashed line indicates the Go cue time. **(B)** Change-point detection applied to the cursor position data. Blue crosses indicate abrupt changes detected using the *ischange* function in MATLAB, and the red curve shows the Gaussian-smoothed trace of the detected change points (mean = 0 ms, SD = 10 ms). The black dashed line denotes the cutoff value (0.05) used to determine state transitions. **(C)** Binary reference labels derived from the smoothed change-point trace by the cutoff value, indicating stationary (0, blue) and movement (2, red) states over time. **(D)** Overlay of the final reference labels on the cursor position data, illustrating the correspondence between labeled states and observed cursor position.

Cursor displacement information was used to create labels for the stationary and movement states. To avoid errors in labeling, for example due to the delayed reaction of the NHPs to task stimuli (as can be seen in Figure 1A), labels were verified manually. The *ischange* function in MATLAB was first applied to detect abrupt changes in the x- or y-position of the cursor, producing a binary vector in which change points were marked as “1.” These markers were then convolved with a Gaussian kernel to obtain a smoothed trace. The final state labels were generated by applying a cutoff value. Gaussian convolution parameters and the cutoff values were manually adjusted for each trial based on manual inspection. The Gaussian kernel standard deviation was 10.3 ± 1.0 ms (mean ± standard deviation) for NHP J and 9.70 ± 1.02 ms for NHP N. The cutoff values were 0.0498 ± 0.0057 for NHP J and 0.0549 ± 0.0088 for NHP N. All adjustments were performed without reference to neural activity or classifier outputs.

An example from Trial 1 of NHP J is shown in Figure 1. The change points detected using *ischange* are marked with blue crosses, and the Gaussian-smoothed trace is plotted in red (mean = 0 ms, SD = 10 ms; Figure 1B). The cutoff used to generate the final labels is indicated by the black dashed line (cutoff = 0.05; Figure 1B). The resulting stationary and movement annotations are displayed in Figures 1C, D. Although manual refinement cannot eliminate all potential misclassifications, it substantially reduces labeling errors.

### 2.3 Comparison group: threshold-crossing method

The threshold-crossing (TC) method (Equation 1) was used as a baseline method for comparison against the proposed scheme: if the velocity of the cursor (x- or y-velocity) is lower than the threshold value, it is recorded as stationary; otherwise, it is recorded as movement. This method was applied to all trials. The performance (accuracy, sensitivity, specificity, precision, and *F*-score) was calculated for each trial after testing.


$$Modified\;output=\{\begin{array}{cc} 0, & Output<Threshold\\ Output, & Output\geq Threshold \end{array}$$ (1)

This is a simple and common way to filter decoder outputs, applicable as a generic add-on to almost any model, ensuring that the effector remains motionless below a certain speed (George et al., 2018, 2020; Page et al., 2018). This solution is flexible and can quickly adapt to task requirements and correct model output. However, its reliance on hard rules reduces generalization ability; rigidly defining a minimum motion speed can lead to misclassification of low-speed motions, causing discontinuities and negatively affecting the user experience.

Importantly, the TC method operates directly on kinematic signals, and its mechanism is determined once the rule is defined, without requiring training or parameter learning as in learning-based models. Accordingly, when used as a comparison group, the TC method is not intended to be evaluated under the same processing workflow as learning-based models, but rather to be regarded as a reference reflecting typical application scenarios in practical BCI pipelines.

### 2.4 New model (correlation-PCA SVM model)

The new model directly classifies stationary and movement states using neural signals. The new classification model (correlation-PCA SVM model; “cpSVM,” Graphical Abstract) consisted of principal component analysis (PCA), correlation analysis, and a Support Vector Machine (SVM) with a linear kernel, feature standardization (z-score normalization), and a default box constraint (C = 1). No class weighting was applied. Model training used the sequential minimal optimization (SMO) solver with default convergence tolerance (eps = 10^−3^), and no manual hyperparameter tuning was performed. For each NHP, the data were reduced in dimension by PCA to reduce the training time of the classifier and speed up classification. PCA alone does not indicate which principal components (PCs) carry task-related information nor does it provide a principled way to determine how many PCs should be retained. The selection of PCs should therefore depend on task-specific characteristics rather than a fixed number.

To maximize the retention of the task-related information during the dimensionality reduction process, correlation analysis was used to select eligible PCs based on the correlation coefficients between the PC projections and the reference labels. PCs were retained if the magnitude of correlation coefficient between their projection and the reference labels was no less than the set value (benchmark). The pattern of combining correlation and PCA is called the cPCA (dash frame in Graphical Abstract). This correlation-based feature selection strategy has also been applied in other domains, such as breast cancer diagnosis in biomedical imaging (Ibrahim et al., 2021) and machine learning applications (Li et al., 2011). Both approaches rely on correlation results to identify features that best capture task-specific or class-discriminative information.

The model was tested with 10-fold cross-validation at the trial level. For each fold, 90% of the trials were used as the training set and the remaining 10% as the test set. The cPCA process was performed using the training set only. After selecting eligible PCs, the SVM classifier was trained using the projections of the selected PCs and the reference labels corresponding to the training data. In the test section, each trial of the test data was projected onto the selected PCs then subjected to the trained SVM classifier to classify motion states. The predictions within each tested trial were aggregated to compute the performance (accuracy, sensitivity, specificity, precision, and *F*-score) for each fold.

## 3 Results

### 3.1 Neural activity and correlation coefficient benchmark analysis

**Figure 2:**
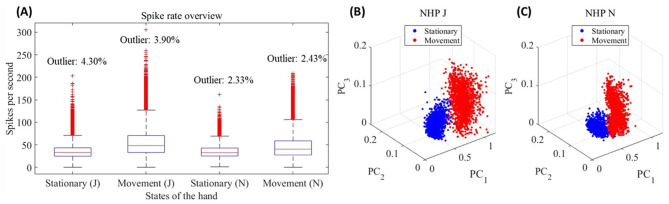
**(A)** Box plot of the spike rate for each state across all channels for all trials of NHPs J and N. **(B)** 3D scatter plot of each trial for NHP J using PCA. **(C)** 3D scatter plot of each trial for NHP N using PCA.

Reference labels identifying stationary and movement states were the basic task information for this study. Since there is a relationship between neural activity and motion (Fuchs et al., 2000; Pujol et al., 2014), task information should also appear in neural activity. Spike rates were calculated for each channel and for each trial for both NHPs J and N. The box plot of the spike rate results after aggregation across arrays A and B is shown in Figure 2A. Spike rates were not normally distributed according to the Shapiro–Wilk test (*P* < 0.001), leading to the use of the Wilcoxon signed rank test to confirm a significant difference between the categories (*P* < 0.001) within each NHP. This result suggested the difference in spike rates between stationary and movement states was a manifestation of task information in neural activity.

Figures 2B, C are D scatter plots using PCA for all trials for NHPs J and N, respectively. The three dimensions represent the projections of the first three principal components without correlation-based selection. These scatter plots clearly show the distinct separation between stationary and movement states, demonstrating the effectiveness of PCA in separating these behavioral states and highlighting differences in neural activity.

**Figure 3:**
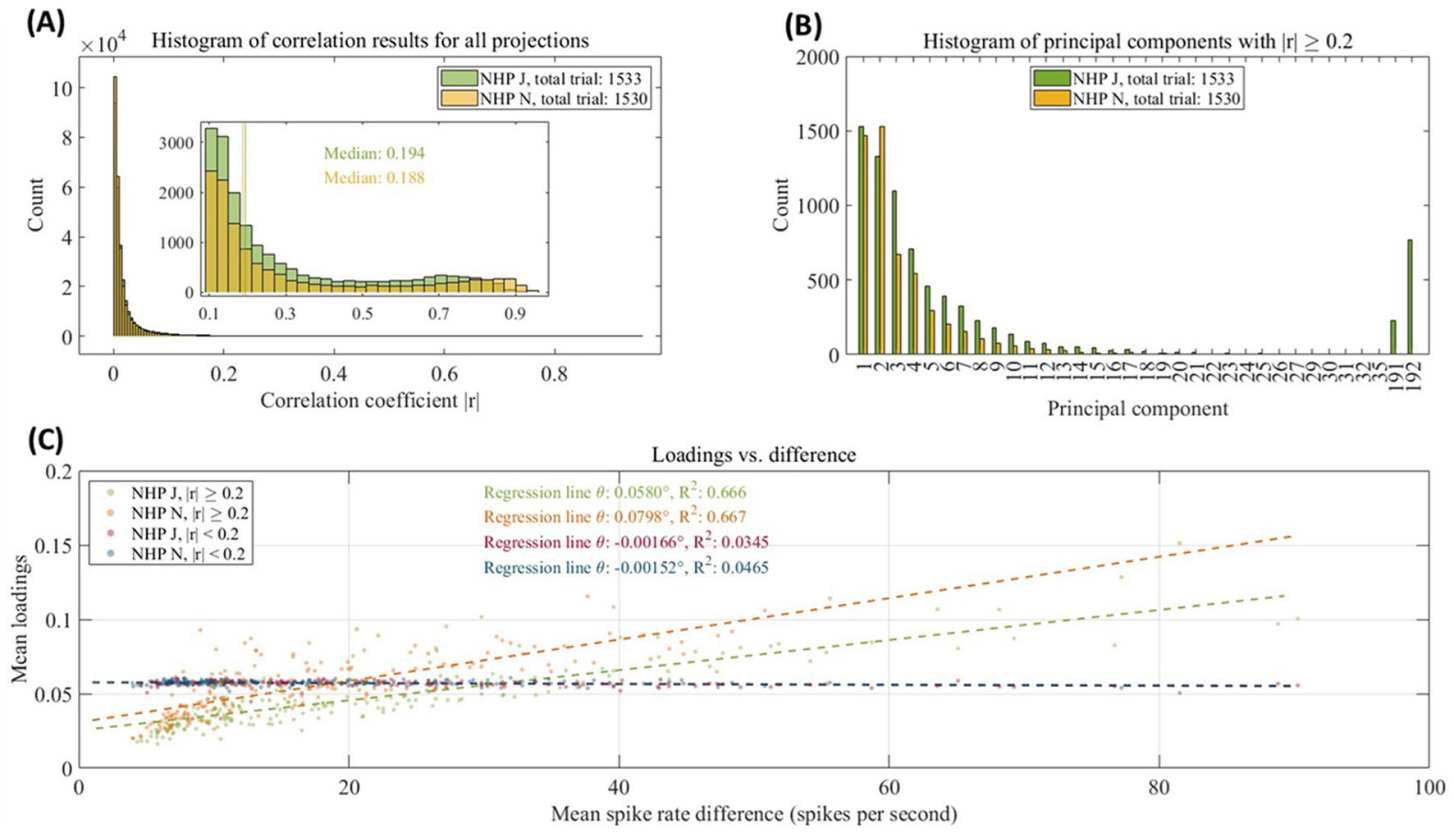
**(A)** Histogram of the correlation coefficients (|*r*|) for principal components (PCs) projections and labels for all trials of NHPs J and N. The vertical line indicates the medians of NHPs J and N in the range 0.1 to 1 of |*r*|. **(B)** Histogram of PCs with |*r*| ≥ 0.2 for all trials of NHPs J and N. **(C)** Scatter plot of the mean loadings and mean spike rate difference per channel for NHPs J and N. The dotted lines are the linear regressions. The slope (angle, Θ) and *R*^2^ of the regression statistics are displayed in the plot.

To retain as much task information as possible, correlation analysis was applied to the PCA results (“cPCA”). Figure 3A shows histograms that summarize the magnitude of correlation coefficients (|*r*|) between all projections and labels from each trial, after applying PCA individually to each trial for NHPs J and N. This analysis was performed on the full dataset to characterize the distribution of correlation coefficients and to establish a global correlation benchmark. Correlations in the |*r*| < 0.1 range were very small and did not show a statistically significant (*P* > 0.001, Pearson correlation), so values this range were excluded from further analysis. Focusing on the range of 0.1–1 (*P* < 0.001, Pearson correlation), the medians for NHPs J and N are 0.194 and 0.188, respectively. Based on these median values and the “weak correlation” criterion (|*r*| ≥ 0.2) defined in Evans (1996), a selection criterion of |*r*| > 0.2 was adopted to identify task-relevant PCs. Figure 3B presents histograms that summarize the PCs with |*r*| ≥ 0.2, selected from each trial using the cPCA, for NHPs J and N.

The loadings of each PC, reflecting the weight or importance of each variable in the PCs, are displayed in Figure 3C as scatter plots of the mean loading and mean spike rate difference per channel for NHPs J and N. Two types of mean loadings were calculated: for all PCs with |*r*| ≥ 0.2, and for all PCs with |*r*| < 0.2. The mean spike rate differences were calculated as the means of the absolute differences in spike rates between stationary and movement states. Linear regressions were performed on these scatter points, and the slope (angle, Θ) and *R*-square of the regression statistics were calculated. The regression results for the PCs with |*r*| ≥ 0.2 showed a steeper slope (Θ > 0.05°) and higher *R*-square (*R*^2^ > 0.6), indicating a strong linear relationship between loadings and spike rate differences, and suggesting that these PCs capture the task information significantly. In contrast, the PCs with |*r*| < 0.2 exhibited nearly flat (Θ ≈ 0°) regression lines with lower *R*-square (*R*^2^ < 0.05), highlighting their minimal contribution to explaining the neural activity related to the task information.

### 3.2 Performance of the cpSVM vs. threshold-crossing method

The 10-fold cross-validation was used to test the performance of the cpSVM (Graphical Abstract). The mean training time per fold was 3.89 ± 0.123 h (mean ± 95% confidence interval), and the mean decode time per prediction was 0.647 ± 0.001 ms, measured using MATLAB on the described hardware setup.

**Figure 4:**
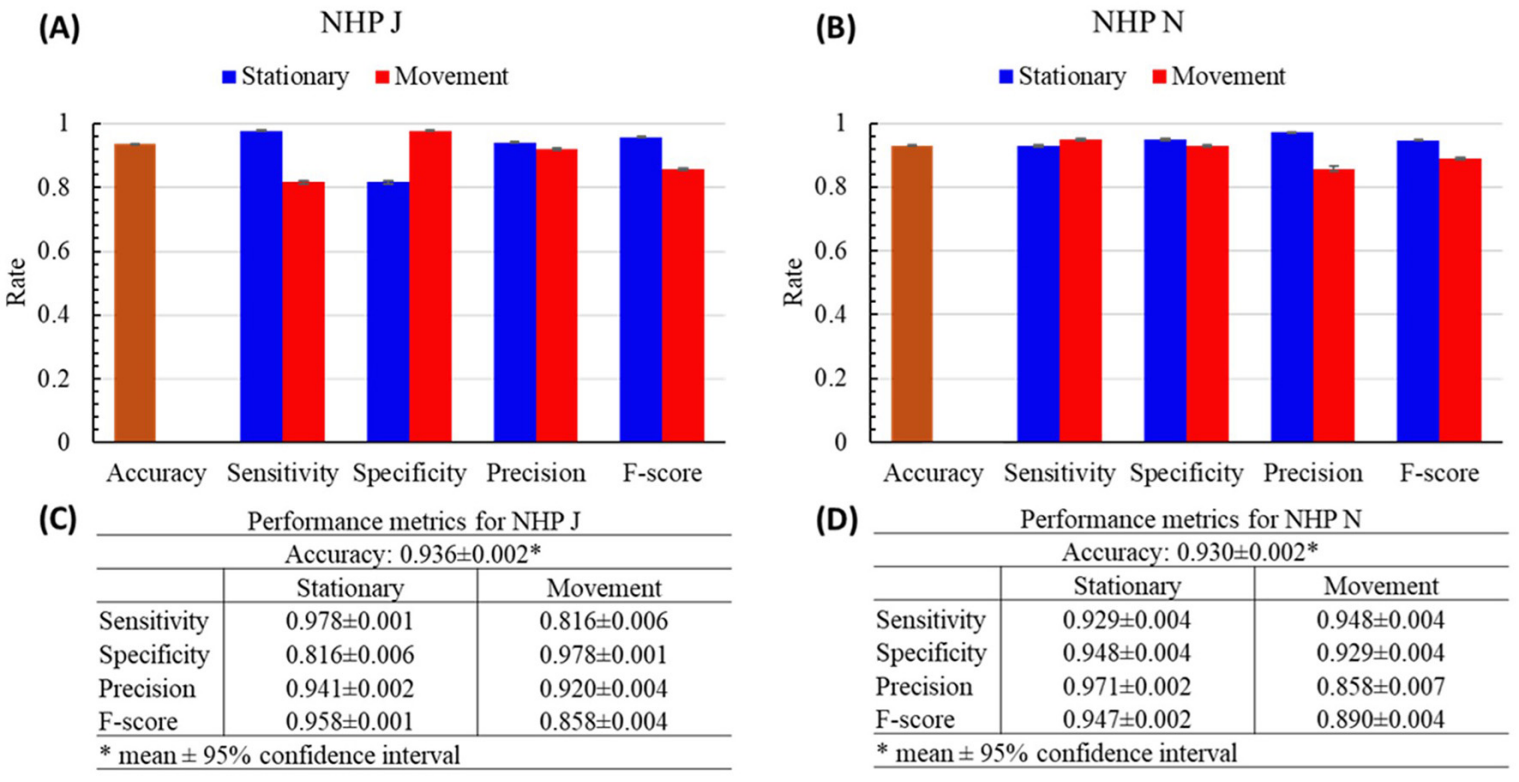
Performance results of the cpSVM with the 10-fold cross validation of NHPs J and N. **(A, B)** The bars are the mean of each performance metric. Error bars are the 95% confidence interval. **(C, D)** The numeric display for panels **(A)** and **(B)**.

The performance (accuracy, sensitivity, specificity, precision, and *F*-score) of each tested fold are shown in Figure 4. The mean accuracies of both NHPs were at or above 0.930 (J: 0.936 ± 0.002; N: 0.930 ± 0.002). The mean values of the other performance metrics were all higher than 0.9, except for the mean sensitivity (0.816 ± 0.006) and *F*-score (0.858 ± 0.004) of the movement state for NHP J; mean specificity (0.816 ± 0.006) of the stationary state for NHP J; and the mean precision (0.858 ± 0.007) and *F*-score of the movement state (0.890 ± 0.004) for NHP N.

The cpSVM was compared with the threshold-crossing (TC) method. After evaluating threshold values between 10 and 40 independently for the x- and y-components of cursor velocity, the highest mean accuracy across all trials was obtained when threshold values of 25 were applied for both components in NHPs J and N. All prediction times were less than 0.1 ms, measured using MATLAB on the described hardware setup, which was faster than the cpSVM.

**Figure 5:**
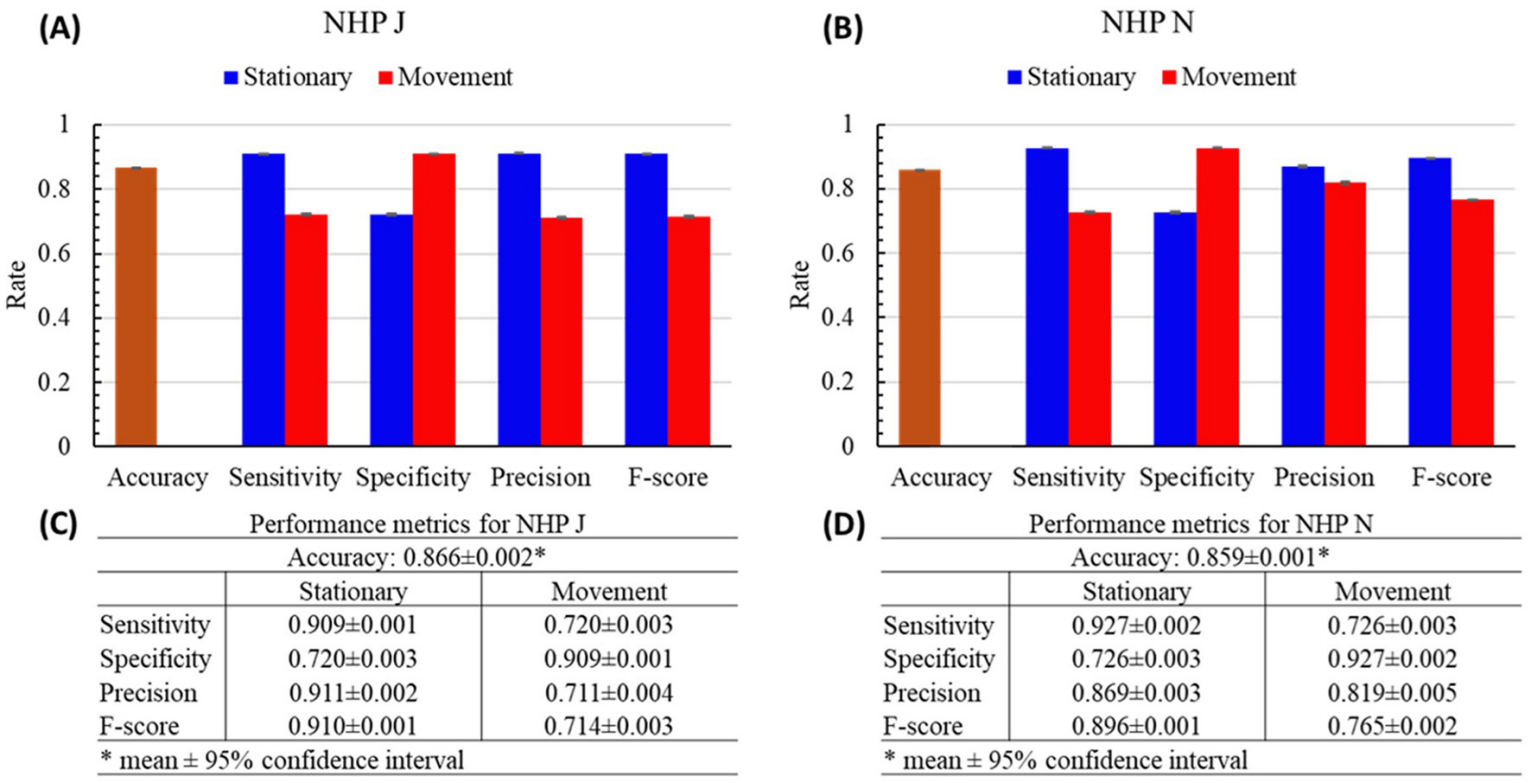
Performance results of the threshold-crossing (TC) method of NHPs J and N. **(A, B)** The bars are the mean of each performance metric. Error bars are the 95% confidence interval. **(C, D)** The numeric display for panels **(A)** and **(B)**.

The performance results of the TC method of each NHP are shown in Figure 5. Compared with the accuracies of the cpSVM, the mean accuracy of NHP J was 0.866 ± 0.002 and that of NHP N was 0.859 ± 0.001, both about 8% lower, indicating that thresholding decoded velocity alone provides noticeably weaker discrimination of stationary vs. movement. Meanwhile, comparing the other performance metrics, all performance metrics of the TC method were lower than the cpSVM. For the stationary state of NHP J, the mean sensitivity was reduced by 7.06%; mean specificity by 11.76%, mean precision by 3.19%, and the mean *F*-score by 5.01%. For the stationary state of NHP N, the mean sensitivity was reduced by 0.215%; mean specificity by 23.4%, mean precision by 10.5%, and mean *F*-score by 5.39%. For the movement state of NHP J, mean sensitivity was decreased by 11.8%; mean specificity by 7.06%; mean precision by 22.7%; and mean *F*-score by 16.8%. For the movement state of NHP N, mean sensitivity decreased by 23.4%; mean specificity by 0.215%; mean precision by 4.55%; and mean *F*-score by 14.0%.

These reductions in performance metrics indicate that the TC method is fundamentally constrained by its dependence on a fixed velocity threshold and exposes its primary failure mechanism. Because decisions rely solely on whether the decoded velocity exceeds this threshold, slow or low-amplitude movements frequently fall below it and are consequently misclassified as stationary. This misclassification pattern increases false positives for the stationary class and false negatives for the movement class, leading to an overall decline in performance. The systematic bias toward the stationary state causes a substantial portion of true movement events to go undetected, leading to lower performance and reduced robustness compared with the cpSVM.

### 3.3 Comparison of transition and responsiveness

**Figure 6:**
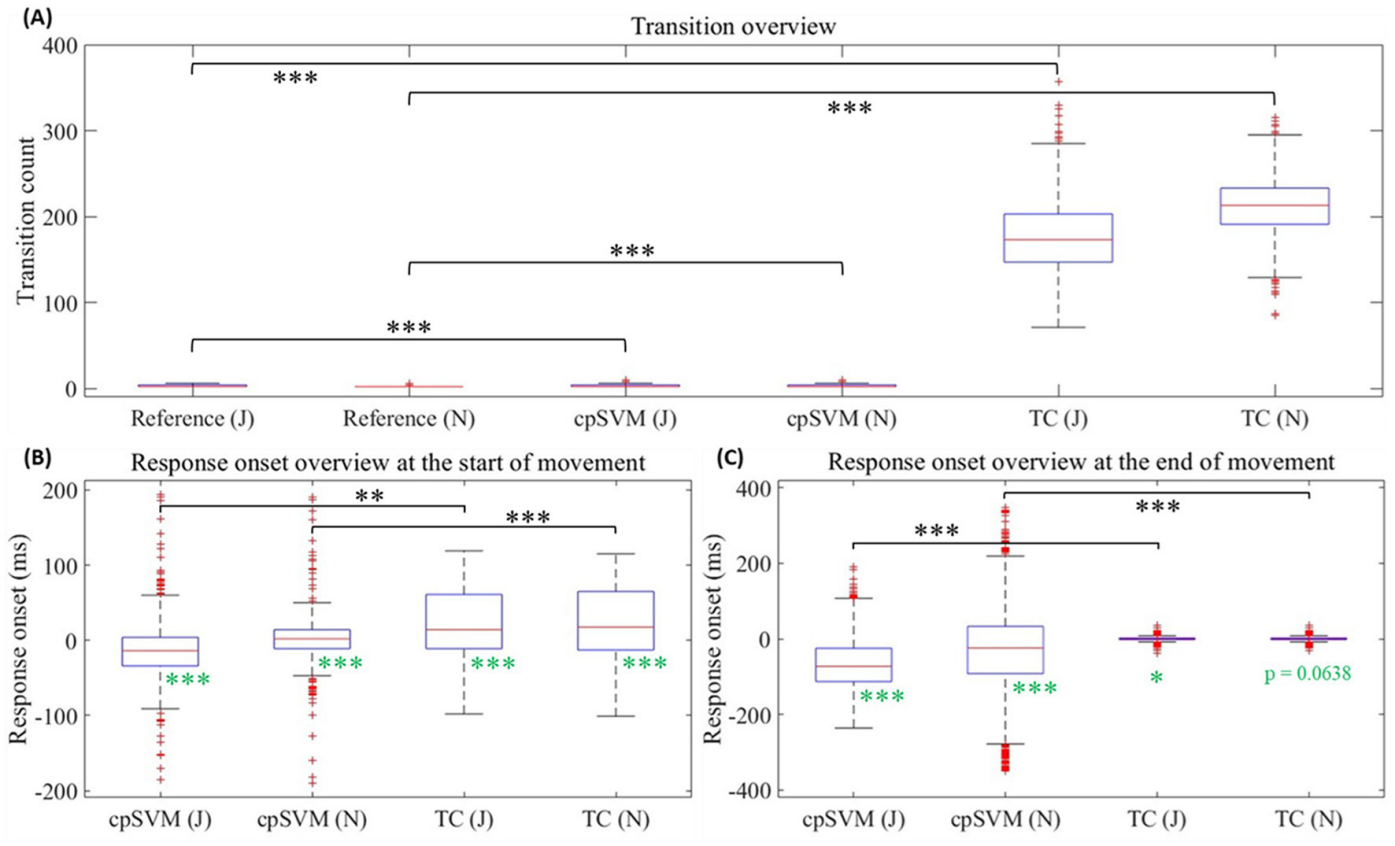
**(A)** Box plot of the transition for the reference label, cpSVM and threshold-crossing (TC) method. **(B)** Box plot of the response onset for the cpSVM and TC method at the start of cursor movement. **(C)** Box plot of the response onset for the cpSVM and TC method at the end of cursor movement. Black brackets indicate paired-sample *t*-tests, and black asterisks indicate their significance levels. Green asterisks indicate the significance levels of one-sided one-sample *t*-tests. Significance levels are denoted as *P < 0.025 (one-sided), **P < 0.01, and ***P < 0.001.

Table 1 and Figure 6A present descriptive statistics of transitions between states for the cpSVM and TC method. A state change, either from stationary to movement (the start of cursor movement) or from movement to stationary (the end of cursor movement), was denoted as a transition. For each trial, there were generally two transitions (J: 2.69 ± 0.05; N: 2.31 ± 0.04). The number of transitions in the cpSVM outputs (J: 3.01 ± 0.07; N: 3.04 ± 0.07) differed significantly from the number of transitions in the reference labels according to paired-sample *t*-tests (J: *t*(1,532) = 8.41, *P* < 0.001; N: *t*(1,529) = 17.8, *P* < 0.001). However, the effect sizes (J: Cohen's *d* = 0.215; N: Cohen's *d* = 0.456) suggest that the difference was small for NHP J and small-to-medium for NHP N, indicating that the number of transitions in the cpSVM outputs remained broadly consistent with the reference labels. In contrast, the TC method had substantially more transitions (J: 175 ± 2; N: 211 ± 1) than the reference labels (J: *t*(1,532) = 204, *P* < 0.001, Cohen's *d* = 5.20; N: *t*(,1529) = 315, *P* < 0.001, Cohen's *d* = 8.06, paired-sample *t*-tests), with extremely large effect sizes.

**Table 1:** Descriptive statistics of the transition metric per trial.

**Method**	**NHP J**	**NHP N**
cpSVM	3.01 ± 0.07	3.04 ± 0.07
TC	175 ± 2	211 ± 1

Counts indicated as mean ± 95% confidence interval.

Table 2, and Figures 6B, C, summarize responsiveness for the cpSVM and TC methods. Responsiveness was characterized by two metrics: reactivity and sensitivity. Response reactivity was calculated as the difference in the time of the decoded state onset relative to the time of the reference label transition, retaining the sign. Negative values of reactivity indicate that the decoded state occurred earlier than the reference label transition and is therefore predictive; positive values correspond to reactive transitions. Response sensitivity was calculated from the absolute value of this same difference. High sensitivity corresponds to a small time delta between the time of the decoded state onset and the time of the reference transition.

**Table 2:** Statistics of the responsiveness per trial.

**Analysis**	**Method**	**Variable**	**NHP J**		**NHP N**	
			**Movement start**	**Movement end**	**Movement start**	**Movement end**
Onset	cpSVM	Time (ms)	−16.5 ± 3.9	−66.7 ± 3.5	−88.7 ± 14.6	−73.6 ± 9.0
		*P*-value^∧^	< 0.001	< 0.001	< 0.001	< 0.001
		Cohen's *d*	−0.212	−0.940	−0.304	−0.410
	TC	Time (ms)	20.0 ± 2.4	0.240 ± 0.235	21.6 ± 2.4	0.182 ± 0.234
		*P*-value^∧^	< 0.001	0.0227	< 0.001	0.0638
		Cohen's *d*	0.418	0.0511	0.453	0.0391
Sensitivity	cpSVM vs. TC	*P*-value^#^	0.00134	< 0.001	< 0.001	< 0.001
	Cohen's *d*	−0.0822	1.36	0.250	0.907

Time indicated as the relative timing of the detection with respect to the reference label state change, mean ± 95% confidence interval.

^∧^P-values were computed from one-sided one-sample t-test on the difference in time between reference label state change and the detection method's response.

^#^P-values were computed from paired-sample t-test on the magnitudes of response onset (absolute values) between the cpSVM and TC method.

Cohen's d represents the effect size, with absolute values of 0–0.2 considered negligible, 0.2–0.5 small, 0.5–0.8 medium, and > 0.8 large. The sign indicates the direction of the difference.

Response reactivity was typically predictive for cpSVM and reactive for the TC method. One-sided one-sample *t*-tests confirmed that the cpSVM exhibited significantly earlier onset for both NHPs J and N, with effect sizes ranging from small to large (J: Start, *t*(1,532) = −8.30, *P* < 0.001, Cohen's *d* = −0.212; End, *t*(1,532) = −36.80, *P* < 0.001, Cohen's *d* = −0.940. N: Start, *t*(1,529) = −11.89, *P* < 0.001, Cohen's *d* = −0.304; End, *t*(1,529) = −16.04, *P* < 0.001, Cohen's *d* = −0.410). In contrast, the TC method exhibited significantly later onset at the start of cursor movement with small-to-medium effect sizes (J: *t*(1,532) = 16.4, *P* < 0.001, Cohen's *d* = 0.418; N: *t*(1,529) = 17.7, *P* < 0.001, Cohen's *d* = 0.453). At the end of cursor movement, NHP J showed a small but significantly later onset (*t*(1,532) = 2.00, *P* = 0.0227, Cohen's *d* = 0.0511), whereas NHP N's response onset was not significantly earlier or later (*t*(1,529) = 1.53, *P* = 0.0638, Cohen's *d* = 0.0391), both with negligible effect sizes.

Paired-sample *t*-tests conducted on response sensitivity between the cpSVM and TC method showed higher sensitivity for the TC method than cpSVM. The sign of the *t*-statistic (and Cohen's *d*) indicated which method yielded smaller absolute deviations, that is, responses closer to zero and therefore higher sensitivity. At the start of cursor movement, NHP J exhibited marginally higher sensitivity for the cpSVM with a negligible effect size (*t*(1,532) = −3.22, *P* = 0.00134, Cohen's *d* = −0.0822), whereas NHP N showed slightly higher sensitivity for the TC method with a small effect size (*t*(1,529) = 9.78, *P* < 0.001, Cohen's *d* = 0.250). At the end of cursor movement, the TC method demonstrated markedly higher sensitivity (J: *t*(1,532) = 53.3, *P* < 0.001, Cohen's *d* = 1.36; N: *t*(1,529) = 35.48, *P* < 0.001, Cohen's *d* = 0.907), both with large effect sizes.

## 4 Discussion

Analysis of neural activity reveals robust differences between behavioral states of movement and non-movement. Figure 2A reveals a significant difference in spike rates between the stationary and movement states, which is confirmed by the Wilcoxon signed rank test. The Figure 2B demonstrate the effectiveness of PCA in distinguishing between stationary and movement states. This clear separation not only validates the efficacy of PCA in analyzing neural data but also highlights the critical role of principal components (PCs) in revealing differences between behavioral states. When setting the correlation coefficient benchmark for the cPCA at 0.2, the comparative analysis of PC loadings and spike rate differences (Figure 3C) indicates that the PCs with |*r*| ≥ 0.2 effectively captured neural changes associated with the task information. These findings highlight that the cPCA not only enhances the relevance of PC selection but also improves the model's explanatory power for neural activity, demonstrating its utility in studying the neural activity underlying specific behavioral states.

The applicability of cpSVM is demonstrated by comparing the performance results of the threshold-crossing (TC) method with those of the cpSVM with the 10-fold cross-validation (Figures 4, 5). Based on the results, the cpSVM outperformed the TC method across all performance metrics. While the TC method achieved faster prediction times, the cpSVM's prediction time remains very short, suggesting that prediction latency is not a limiting factor. Such results show that the cpSVM was able to separate the stationary and movement states effectively.

Other metrics such as transition count and responsiveness (Tables 1, 2 and Figure 6) need to be considered for user experience and safety. The TC method might exhibit hundreds of transitions in a trial whereas the cpSVM has substantially fewer transitions. Frequent transitions can lead to a decrease in the continuity of motion, causing unintended cursor movement during intended non-movement or interruptions to intended cursor movement. In comparison, the cpSVM has better continuity than the TC method.

**Figure 7:**
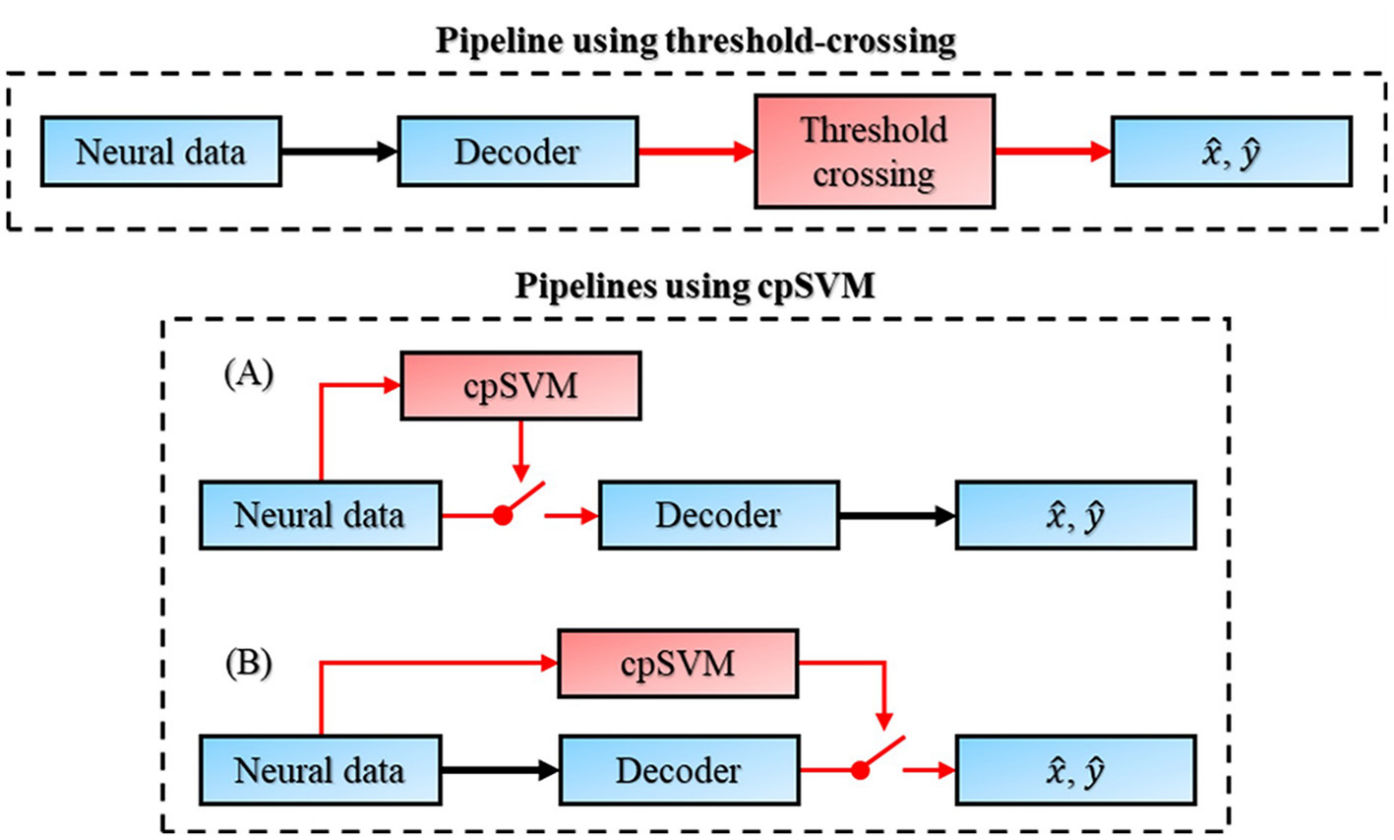
Pipelines of using the threshold crossing method or cpSVM method in motor decoding BCI applications.

In terms of the response reactivity, the cpSVM's onset generally occurred before the reference label state change, whereas the TC method's onset occurred after. The difference of the response onset reflects the mechanism underlying each method: the TC method relies on behavioral observation whereas the cpSVM evaluates neural activity that generally leads to behavior (Figure 7). Because of the inherent delay between neural activity and physical response, the cpSVM's onset is about 16.5–88.7 ms earlier than the reference label state change. Although response sensitivity was worse for the cpSVM method, its predictive timing relative to response onset alleviates its larger magnitude sensitivity (Table 2, Figures 6B, C). Such early responses highlight the predictive nature of the cpSVM and can be advantageous in BCI applications by allowing earlier detection of intended movements.

The advantage of the TC method is that its algorithm is simple, and it only needs to set a threshold for the output to clear the unintended trajectories. However, it suffers from lower performance and may lead to discontinuities in motion. In comparison, cpSVM has two main disadvantages: its algorithm is more complex and consumes more computational power during training, and it is less sensitive in responsiveness than the TC method, which may result in a less seamless user experience. Nonetheless, the cpSVM retains several advantages, including higher classification performance, smoother state transitions, and a predictive capability that anticipates movement intention, thereby offering a more reliable control for BCI applications.

cpSVM itself is just a plug-in gating module to the whole BCI system (Figure 7) and should not alter the core decoding or encoding processes, allowing it to be applied to most movement decoders or replaced with other feasible models. Although placing the cpSVM after the decoder (Figure 7B) is possible, this configuration introduces several limitations. Because the movement decoder will continuously process neural inputs, such a downstream placement increases computational load and energy consumption, and can cause the internal decoder state to drift when driven by neural activity during stationary. For recurrent neural decoders, updating with neural activity that is not associated with movement may perturb or destabilize their latent space dynamics. For filter-based decoders, such as Kalman filters, the propagation of movement-unrelated neural activity can lead to undesirable updates of internal parameters (e.g., the Kalman gain), thereby reducing decoding stability. Similar drift can also affect adaptive decoders, which update parameters online and may adapt to neural fluctuations during stationary. Together, these effects can degrade decoder stability and reduce the reliability of BCI control over time. In contrast, placing the classifier before the decoder (Figure 7A) establishes an upstream gating mechanism can avoid these effects by blocking neural inputs during stationary, thereby helping to maintain decoder stability and reducing unnecessary computational and energy costs.

There are two research directions that can make use of this model. One is to better support precision-critical applications, for example, the use of neural signals to stimulate muscles at the site of paralysis (Milosevic et al., 2020a,b; Osuagwu et al., 2016) or to control a rehabilitation device (Bhagat et al., 2020; Cheng et al., 2020; Robinson et al., 2021; Wu et al., 2020). In these systems, small mismatches between neural signals and the user's intended behavioral state can lead to inaccurate stimulation timing or erroneous device activation, ultimately impairing motor recovery or functional control. A reliable classifier can therefore help ensure that stimulation or device actuation occurs only when a movement is truly intended.

Another is to support the safety-critical applications such as wheelchair (Espiritu et al., 2019; Lahane et al., 2018; Tang et al., 2018) or vehicle control (Dunlap et al., 2019; Xiao et al., 2025). In these scenarios, preventing unintended commands is essential, as even small false-positive activations can result in hazardous movements. Therefore, eliminating unnecessary behavioral trajectories can enhance both accuracy and controllability of the BCI systems, thereby improving overall safety.

While these results are promising, there are several limitations to this study. First, the analyses were conducted entirely offline, and the proposed method was not evaluated within a complete online decoder or closed-loop BCI system. As such, its real-time performance and impact on control stability remain to be validated. In addition, the predictive behavior of the cpSVM, may require task- and application-specific considerations to avoid unintended decoder output during preparatory periods in closed-loop settings. Furthermore, the dataset was restricted to recordings from the PMd and M1 of two NHPs performing a specific reaching task, which may limit the generalizability of the findings to other cortical regions, tasks, or subject populations. Moreover, the creation of reference labels relied on manually chosen Gaussian kernel and cutoff parameters, these steps may introduce subjectivity and residual misclassification, which require tuning for different datasets. Finally, the method was tested only on multi-unit activity, and its applicability to other neural signal modalities (e.g., single-unit activity, local field potentials) remains to be explored.

Ongoing work and future plans include implementing the proposed approach with continuous movement decoders in a closed-loop BCI setting, where its effects on trajectory-level continuity and user experience can be directly evaluated under realistic control conditions. Additional studies will involve analyzing datasets from different subjects, tasks, and cortical regions, and exploring other feasible models for movement classification and continuous decoding. These could include probabilistic approaches such as hidden Markov models (HMMs) and deep learning architecture (e.g., long short-term memory networks, convolutional neural networks, etc.). Future studies should also evaluate the robustness of the method across long-term recordings and its applicability to other neural signal modalities.

## 5 Conclusion

For BCI applications, decoders have shown good performance in their ability to decode neural signals into motion trajectories; however, during periods of no physical movement, neurons are still firing, so the decoders may output unintended commands. This paper introduces the cpSVM model that classifies intended stationary and movement states of the controlled effector using neural activity. In comparison with the threshold-crossing method, a simple and common way to filter decoder outputs, cpSVM performs better in terms of accuracy, sensitivity, specificity, precision, *F*-score, and continuity, which may provide a new alternative solution to achieve a more reliable control for BCI applications.

## Data Availability

Publicly available datasets were analyzed in this study. This data can be found here: https://dandiarchive.org/dandiset/000070.
